# Supplementary material for: NSD2 is a requisite subunit of the AR/FOXA1 neo-enhanceosome in promoting prostate tumorigenesis
Source: Nat Genet. 2024 Sep 9;56(10):2132–43. doi: 10.1038/s41588-024-01893-6 (PMC11525188; doi:10.1038/s41588-024-01893-6)
Supplement: Supplementary file 1 — Supplementary Information. [file 41588_2024_1893_MOESM1_ESM.pdf]

nature genetics

Supplementary information

https://doi.org/10.1038/s41588-024-01893-6

NSD2 is a requisite subunit of the AR/FOXA1 neo-enhanceosome in promoting prostate tumorigenesis

In the format provided by the authors and unedited

NSD2 is a requisite subunit of the AR/FOXA1 neo-enhanceosome in promoting prostate tumorigenesis

Abhijit Parolia[1,2,3,4,#], Sanjana Eyunni[1,2,5,13], Brijesh Kumar Verma[6,13], Eleanor Young[1], Yihan Liu[1,7], Lianchao Liu[8], James George[1], Shweta Aras[6], Chandan Kanta Das[6], Rahul Mannan[1,2], Reyaz ur Rasool[6], Erick Mitchell-Velasquez[6], Somnath Mahapatra[1,2], Jie Luo[1,2], Sandra E. Carson[1], Lanbo Xiao[1,2], Prathibha R. Gajjala[1,2], Sharan Venkatesh[6], Mustapha Jaber[1], Xiaoju Wang[1,2], Tongchen He[1], Yuanyuan Qiao[1,2], Matthew Pang[1], Yuping Zhang[1,2], Jean Ching-Yi Tien[1,2], Micheala Louw[2], Mohammed Alhusayan[6], Xuhong Cao[1,2,9], Omid Tavana[10], Caiyun Hou[8], Zhen Wang[8], Ke Ding[8], Arul M. Chinnaiyan[1,2,3,4,9,#], and Irfan A. Asangani[6,11,12,#].

INVENTORY

This article has five main figures, ten extended data figures and five supplementary tables. These are listed below:

Extended Data Figure 1-3 are related to Figure 1
Extended Data Figure 4 is related to Figure 2
Extended Data Figure 5-7 are related to Figure 3
Extended Data Figure 8-10 are related to Figure 4

Supplementary Table 1: List of known motifs enriched in the NSD2-dependent AR elements along with their HOMER statistics.
Supplementary Table 2: List of known motifs enriched in the NSD2-independent AR elements along with their HOMER statistics.
Supplementary Table 3: Antiproliferative half-maximal inhibitory concentration (IC50) of LLC0150 across human-derived normal and cancer cell lines.
Supplementary Table 4: NSD2 signature genes and GREAT genes associated with NSD2-dependent and NSD2-independent AR binding sites.
Supplementary Table 5: Primers, siRNA and sgRNA sequences used in this study.

Source Data Figure 1-4: These files have source data for all the applicable main figure panels.
Source Data Extended Data Figure 1-10: These files have source data for all the applicable panels in the extended figure.
Source Data Uncropped Blots: This file provides complete immunoblots for panels in main and extended data figures.

Supplementary Note 1: LLC0150 synthesis and LNCaP-mCherry-PSA reporter cell generation.
Supplementary Note 2: NSD1 and NSD2 function in hematological cancers
Supplementary Note 3: Additional methods and materials

Supplementary Note 1: LLC0150 synthesis and LNCaP-mCherry-PSA reporter cell generation

Synthesis of LLC0150

General information: All chemicals and solvents were obtained from commercial suppliers and used without further purification. Purification was performed using combi-flash Nextgen300. All reactions were monitored by TLC, using silica gel plates with fluorescence F254 and UV light visualization. 1 H NMR spectra was recorded on a Varian Mercury Plus at 400 MHz, and 13C NMR spectra was recorded on a JEOL-ECZ-400S spectrometer at 100 MHz. Coupling constants (J) are expressed in hertz (Hz). Chemical shifts (δ) of NMR are reported in parts per million (ppm) units relative to internal control (TMS). Signal splitting patterns are described as singlet (s), doublet (d), triplet (t), quartet (q), multiplet (m), broad (br), or a combination thereof. The low resolution of ESI-MS was recorded on an Agilent-6120 and the high-resolution mass (resolution-70000) for compound was generated using Q-Exactive Plus orbitrap system, (Thermo Scientific) using electrospray ionization (ESI). Melting point was recorded in Stuart instrument, model smp30. Specific optical rotation was recorded in an Anton Paar MCP 5100 instrument. HPLC was recorded with a Waters 2696, and the column used was a YMC Triart C-18 EXRS (150*4.6) mm 5µm using 0.01M ammonium acetate in (Aq); Mobile phase-B: ACN 100%; Method -T/%B: 0/10, 2/10, 5/85, 13/85, 14/10, 15/10 method and flow rate: 1.0 ml/min. FT-IR was recorded using PerkinElmer Spectrum.

Abbreviations used: DMSO for dimethylsulfoxide, DIPEA for N, N-diisopropylethylamine, MeOH for methanol, DMF for N, N-dimethylformamide, HATU for 1- [bis(dimethylamino) methylene]-1H-1,2,3-triazolo[4,5-b]pyridinium 3-oxid hexafluorophosphate, DCM for dichloromethane, Pd(dppf)Cl2 for [1,1′-bis(diphenylphosphino)ferrocene]dichloropalladium(II)

Synthesis of *N*-cyclopropyl-*N*-(4-((4-((4-((2-(2,6-dioxopiperidin-3-yl)-1,3-dioxoisoindolin-5-yl)amino)butyl)carbamoyl)phenyl)carbamoyl)benzyl)-3-oxo-3,4-dihydro-2H-benzo[*b*][1,4]oxazine-7-carboxamide (LLC0150).

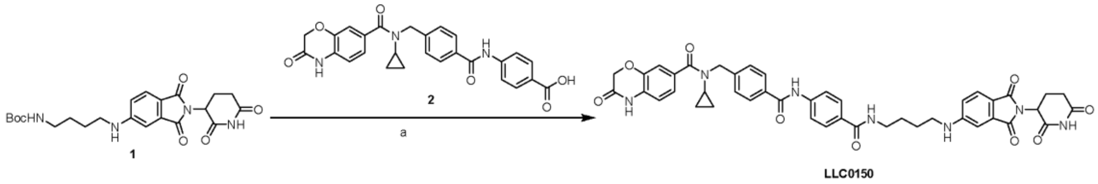

Reagents and conditions: (a) 1) trifluoroacetic acid (TFA), CH_2Cl_2, room temperature (rt), 2 h; 2) **2**, HATU, Et_3N, DMF, rt, 3 h, 83% (two steps).

tert-butyl(4-((2-(2,6-dioxopiperidin-3-yl)-1,3-dioxoisoindolin-5-yl)amino)butyl)carbamate **1** (40.0 mg, 0.0900 mmol) was dissolved in 2.0 mL of CH_2Cl_2. To above solution was added TFA (0.5 mL) by syringe. The mixture was stirred at room temperature for two hours. After the reaction was complete, the solvent was removed by vacuum. The crude product was dissolved in a small amount of DMF (2.0 mL) and were added 4-(4-((*N*-cyclopropyl-3-oxo-3,4-dihydro-2H-benzo[*b*][1,4]oxazine-7-carboxamido)methyl)benzamido)benzoic acid **2** (48.1 mg, 0.0990 mmol),

triethylamine (27.3 mg, 38 μL, 0.270 mmol), and HATU (51.3 mg, 0.135 mmol). The mixture was stirred at room temperature for three hours. The resulting mixture was purified by column chromatography to afford the title compound as a yellow solid (60.6 mg, 83% yield). ^{1}H NMR (600 MHz, DMSO-d_6) δ 11.05 (s, 1H), 10.88 (s, 1H), 10.43 (s, 1H), 8.40 (t, *J* = 5.6 Hz, 1H), 7.96 (d, *J* = 8.2 Hz, 2H), 7.88 – 7.82 (m, 4H), 7.55 (d, *J* = 8.4 Hz, 1H), 7.46 (d, *J* = 7.7 Hz, 2H), 7.22 – 7.13 (m, 3H), 6.98 – 6.95 (m, 1H), 6.93 (d, *J* = 8.1 Hz, 1H), 6.86 (dd, *J* = 8.4, 1.9 Hz, 1H), 5.02 (dd, *J* = 12.8, 5.5 Hz, 1H), 4.72 (s, 2H), 4.62 (s, 2H), 3.30 (d, *J* = 6.2 Hz, 2H), 3.23 – 3.19 (m, 2H), 2.90 – 2.84 (m, 1H), 2.83 – 2.77 (m, 1H), 2.62 – 2.51 (m, 2H), 2.02 – 1.96 (m, 1H), 1.67 – 1.59 (m, 4H), 0.58 – 0.52 (m, 2H), 0.50 – 0.45 (m, 2H); ^{13}C NMR (151 MHz, DMSO-d_6) δ 172.82, 170.19, 167.71, 167.15, 165.64, 165.57, 164.85, 154.46, 142.49, 142.35, 141.70, 134.22, 133.42, 131.79, 129.49, 128.39, 128.06, 127.83, 127.21, 125.10, 121.87, 119.34, 115.81, 115.36, 115.16, 66.74, 48.61, 42.23, 40.06, 38.77, 30.98, 26.82, 25.75, 22.23, 9.55; HRMS (m/z): [M + Na]+ calcd for $C_{44}H_{41}N_7O_9Na^+$ 834.2858, found 834.2861; HPLC purity: 97.92%.

^{1}H NMR of (LLC0150)

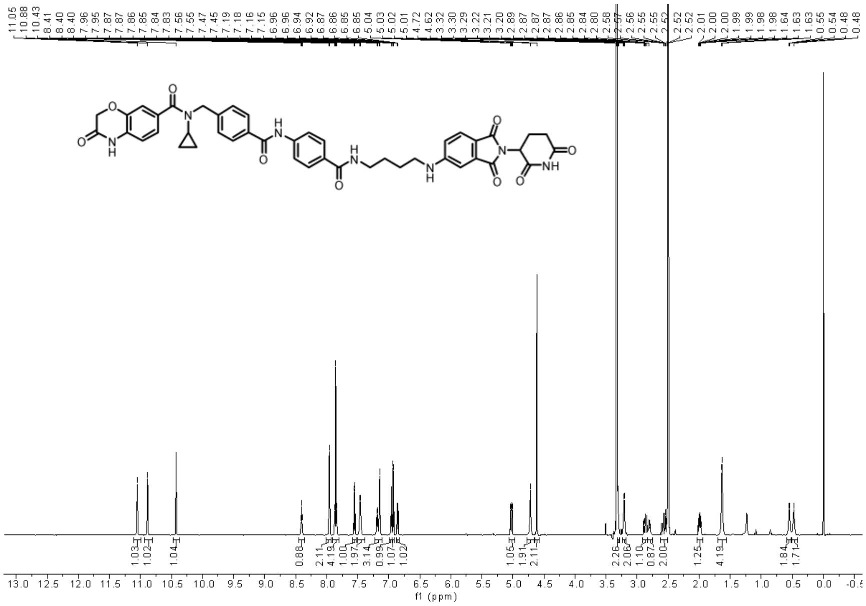

^{13}C NMR of (LLC0150)

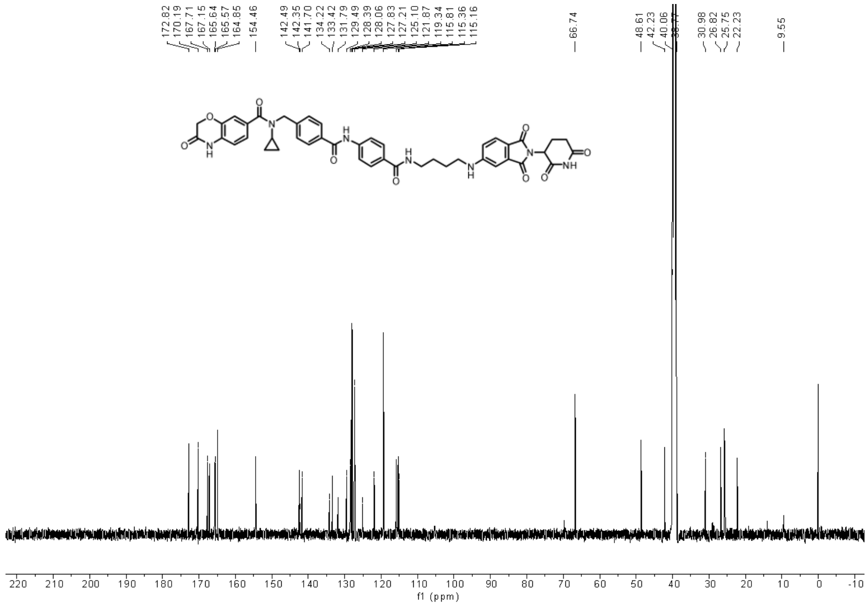

Plasmids and lentivirus

The pMSCV-HA-NSD2-PH WT and pMSCV-HA-NSD2-PH-W236A, pMSCV-HA-NSD2-PH-F266A, pMSCV-HA-NSD2-PH-W236A/F266A, pMSCV-HA-HMGa, pMSCV-HA-HMGa-F463A, pMSCV-HA-HMGa-W491A, and pMSCV-HA-HMGa-Y502A constructs were created through site-directed mutagenesis. Site-directed mutagenesis was performed using the QuikChange II XL site-directed Mutagenesis Kit (Agilent Technologies, 200521) as per the manufacturer's protocol. All mutations were verified by Sanger sequencing. The following primers from Integrated DNA Technologies (IDT) were used for site-directed mutagenesis and cloning:

<u>Site-directed mutagenesis:</u>

pMSCV-NSD2-HMGa-F463A: F-5’-CCAT CCC TGT GTT TTT GAC AGG CGA CCA AAA ACT GGG ATG CTG-3’, R-5’-CAG CAT CCC AGT TTT TGG TCG CCT GTC AAA AAC ACA GGG ATG-3’; pMSCV-NSD2-HMGa-W491A: F-5’-CTC ACT CAG CAG ACT CGC CTG TGA CCT GAG CAG C-3’, R-5’-GCT GCT CAG GTC ACA GGC GAG TCT GCT GAG TGA G-3’; pMSCV-NSD2-HMGa-Y502A:F-5-’GGG CAA ACT TGG TGT TGG CGC GTG CTC TCT GCT TC-3’, R-5’-AGA AGC AGA GAG CAC GCG CCA ACA CCA AGT TTG CCC-3’

<u>Cloning:</u>

pMSCV-HA-NSD2-Fl: F-5’AAG GAT CCG AAT TTA GCA TCA AG-3’, R-5’-TTC TCG AGC TAT TTG CCC TCT GTG AC-3’; pMSCV-HA-NSD2-PH: F-5’-AAG GAT CCG AAT TTA GCA TCA AG-3’, R-5’-TTC TCG AGT TAT CGC TCA GAC TTT TTG GA-3’; pMSCV-HA-NSD2-PH: F-5’-CAA ATG TTG CTT GTC TGG TG-3’, R-5’-GTC AGT CGA GTG CAC AGT TT-3’; pMSCV-HA-NSD2-HMGa: F-5’-TTC TCG AGT TAA TGG AGG AGC GGA AAG CCA AGT T-3’. R-5’-TTC TCG AGT TAT CGC TCA GAC TTT TTG GA-3’; pMSCV-NSD2-HMGa-Y502A:F-5’GGG CAA ACT TGG TGT TGG CGC GTG CTC TCT GCT TC-3’, R-5’AGA AGC AGA GAG CAC GCG CCA ACA CCA AGT TTG CCC-3’;pMSCV-HA-NSD2-PWWP1a: F-5’- AAG GAT CCG AAT TTA GCA TCA AGC AG-3’, R-5’-TTC TCG AGT TAC CTT CTC TTC ATG GGA AT-3’;pJS60-FKBP5-HA-NSD2-Fl: F-5’-GGTGTCGTGACGCGGATGGAATTTAGCATCAAGCAGAGTCC-3’,R-5’-

TTCCACCTGCACTCCTTTGCCCTCTGTGACTCTCCG-3';pJS60:F-5'-CCGCGTCACGACACCT-3',R-5'-GGAGTGCAGGTGGAAACCA-3';pCDNA4c:F-5'-GCG GCC GCT CGA GTC-3', R-5'-GGA TCC TGG TAC CTT ATC GTC AT-3';NSD2-HMGa: F-5'-AAG GTA CCA GGA TCC ATG CGG AGG GCC AAA CT-3', R-5'TCG AGC GGC CGC CTA TCG CTC AGA CTT TTT GGA TGA GAC AG-3'

Other plasmids used in this study were pMSCV-HA-NSD2-FL, pJS60-FKBP5-HA-NSD2-FL, pCDNA4c-HMGa, pFN21K-Halo-AR-FL, pFN21K-Halo-AR-NTD, pFN21K-Halo-AR-DBD, and pFN21K-Halo-AR-HBD. The plasmids were transfected into HEK293-T or LNCaP cells using Lipofectamine 2000 (Invitrogen) as per the manufacturer's protocol. The cells were then harvested 48/72 h post-transfection and subjected to immunoprecipitation or immunoblotting or used for lentivirus production. Lentiviral particles were generated in the lab at the University of Pennsylvania or in collaboration with the Vector Core at the University of Michigan. Following lentiviral production, viral particles were incubated on cells in penicillin/streptomycin-free media for 24 h, followed by media replacement with normal media. Overexpression and knockdown of proteins was confirmed by immunoblotting.

Generation of the endogenous AR reporter and functional CRISPR screen

LNCaP cells were treated with CRISPR guides targeting the promoter-5'UTR junction of *KLK3/PSA* and a knock-in repair template containing the mCherry coding sequence, P2A cleavage site (as represented in **Fig. 1** and **Fig. S1**) along with 750bp of flanking *KLK3* homologous sequences were transfected at a ratio of 1:3 (guide: repair-template) using Lipofectamine 3000 reagent (Thermo Fisher Scientific, Cat# L3000001). To ensure preferential homologous recombination, non-homologous end joining repair (NEHJ) was inhibited using SCR7 (2uM for 24 hours). Post transfection, cells were subject to puromycin selection for three days, and surviving mCherry-positive cells were FACS sorted to establish monoclonal reporter lines. The top 75% of mCherry-positive cells were sorted to ensure robust and uniform mCherry expression. All intended repair junctions were amplified and Sanger sequenced to confirm sequence integration.

KLK3/PSA gRNA sequence used is as follows: 5'-CACCGAAGACAACCGGGACCCACA-3'

Two monoclones, hereafter named as Clone 1 and 2, were used for the downstream CRISPR screen with stable viral integration of the active Cas9 enzyme. Briefly, the cells were treated with an epigenetic guide library (a kind gift from Dr. Christopher Vakoc), puromycin selected, and incubated for eight days, after which they were stimulated with 1nM DHT for 16 hours and sorted into mCherry high and low populations. Input fractions were also collected at Day 3 post-puromycin selection for guide enrichment analysis. Genomic DNA from input and sorted cells was extracted (DNeasy Blood and Tissue kit), and integrated guide RNA sequences were amplified using common primers, with the resulting amplicon pool submitted for next-generation sequencing.

Supplementary Note 2: NSD1 and NSD2 function in hematological cancers

NUP98-NSD1 activating fusions are recurrently found in childhood acute myeloid leukemias[1], while NSD2 translocations and/or catalytic-domain hotspot mutations are detected in multiple myeloma[2–4] and pediatric acute lymphoblastic leukemia[5–7], respectively. In these tumors, NSD2 hyper-activation leads to a global increase in H3K36me2 levels and oncogenic remodeling of the epigenome through direct antagonism of the repressive PRC2/EZH2 machinery[8–10]. The structural basis for this antagonism was recently identified in di/tri-methylation at H3K36 allosterically hindering the loading of H3K27 residue into the catalytic pocket of EZH2 enzyme[11].

Supplementary Note 3: Additional methods and materials

GREAT and Enrichr Analyses – ChIP-seq binding sites

The strongest peaks as determined by MACS peak score, were selected for sites exclusive to the knockout or the control, as well as the shared sites, 1000 from each. We used the webtool GREAT (v4.0.4)[12,13] to generate lists of the genes nearest to these peaks with max extensions iteratively set to 50kb, 100kb, 250kb, and 500kb (settings: hg38, proximal 5kb, 1kb downstream, distal 50-500kb). These genes selected by GREAT were then further examined as input to our RNAseq pipeline for GSEA and additional analysis using curated datasets (as described in RNA-seq methods above). Functional pathway enrichment analysis was performed using a web-based, interactive tool called Enrichr[14] (http://amp.pharm.mssm.edu/Enrichr/). Briefly, GREAT gene lists were provided to Enrichr, following which, specific libraries were selected to identify either 1) transcription factors that were enriched for the target genes in our input list, 2) enriched pathways using KEGG, Reactome, and BioCarta, 3) Gene ontologies. For instance, within their transcription factor database, we selected pathway enrichments that were derived from distinct datasets (ChEA2022, ENCODE histone modifications,etc). Individual gene sets were viewed in Appyter, and high resolution .svg files were used to plot figures. Statistically significant pathways (using the Fisher's exact test) are colored and highlighted in the figures.

DepMap analysis

To perform the DepMap analysis, we used a BioConductor Cancer Dependency map R data package (version 1.18.0). This package can directly access the Broad Institute's CRISPR Achilles DepMap portal. A detailed guide and code to using this package is provided in the reference paper. Briefly, we inputted the CRISPR data using the EH3797 experimental hub. Utilizing the dependency score option, we plotted the rank/location of our gene of interest against a line of common essential genes (red line in our plot) for that lineage. The red line indicates pan-essentiality[15].

Immunofluorescence multiplexing and signal quantification

Multiplexing immunofluorescence (IF) was performed consecutively as NSD2, CK8, and AR monoclonal antibodies described above with each antibody application and signal development kit followed by a denaturation cycle using high temperature and stringency wash to strip off the previous primary and secondary antibody detection system. The sequence of antibody application and the sequence of fluorophores were based on multiple denaturation and stripping optimization steps on contextual positive and negative control tissues. The consecutive IF detection system

for the above-mentioned sequence of antibodies was developed by using the Discovery FITC kit (catalog no. 760-232, Roche-Ventana), Discovery Red 610 (catalog no. 760-245), and the Discovery Cy5 kit (catalog no. 760-238, Roche-Ventana) with DAPI (Prolong gold anti-fade, catalog no. P36931, Invitrogen/Fischer-Scientific). The multiplex staining was evaluated under confocal LMS900 system by Carl Zeiss.

Quantification of IF and IHC signal:
IF: ImageJ was used to quantify the IF signal. Briefly, the color adjustment was done uniformly on all images being analyzed. Colors were converted to an RGB format, and a uniform color threshold was applied to all images. Using the particle measurement options, the integrated density values were obtained. A normalized fluorescence value was calculated and plotted in GraphPad Prism to normalize the signal from different-sized nuclei. The normalized fluorescence value or corrected total cell fluorescence (CTCF) was calculated as[34]:

Integrated Density – (Area of selected cell X Mean fluorescence of background readings)

IHC: For NSD2 IHC quantification, the total number of immune-positive cells out of 100 (n/100) in four representative tumor cells and matched normal prostate epithelial cell regions was recorded and expressed as a percentage of positive cells for quantification purposes.

TUNEL staining: FFPE tissue sections were deparaffinized and rehydrated using xylene and ethanol gradients. TUNEL signal was stained by the In Situ Cell Death Detection Kit, TMR red (Catalog no. 12156792910) according to manufacturer's instruction. TUNEL signal was visualized and imaged using a fluorescence microscope (Zeiss).

Western blot and immunoprecipitation
Cells were lysed in RIPA buffer (ThermoFisher Scientific) supplemented with cOmpleteTM protease inhibitor cocktail tablets (Sigma-Aldrich). Protein concentrations were measured using Pierce BCA Protein Assay Kit (ThermoFisher Scientific). For immunoblotting, equal amounts of protein were resolved in either a NuPAGE 3 to 8%, Tris-Acetate Protein Gel (ThermoFisher Scientific) or NuPAGE 4 to 12%, Bis-Tris Protein Gel (ThermoFisher Scientific) and incubated overnight with primary antibodies. Following incubation with HRP-conjugated secondary antibodies (BioRad), membranes were imaged on an Odyssey CLx Imager (LiCOR Biosciences).

For immunoprecipitation (IP) experiments, nuclear or whole-cell protein extracts were obtained from cells using NE-PER nuclear extraction kit (Thermo Scientific, 78835) or RIPA buffer, respectively. The nuclear pellet was then lysed in an IP buffer by sonication. Nuclear lysates (0.5-2.0 mg) were pre-cleared by incubation with protein G Dynabeads (Life Technologies, 10003D or Thermo Scientific, 14321D) for 1 h on a rotator at 4°C. Next, an antibody (2-5 µg) was added to the pre-cleared lysates and incubated on a rotator at 4°C overnight. The following day, Protein G Dynabeads were added to the nuclear lysate-antibody mix for 1 h. Beads were washed twice in IP or RIPA buffer containing 300 mM NaCl, resuspended in 40 µL of 2x loading buffer, boiled at 100°C for 5 min, and subjected to SDS-PAGE and immunoblotting. For Halo-tag immunoprecipitation, the cells were lysed in NP-40 buffer (0.1% NP-40, 15 mM Tris–HCl pH7.4,

1 mM EDTA, 150 mM NaCl, 1 mM MgCl2, 10% Glycerol) containing protease and phosphatase inhibitors and the lysates were incubated with anti-Halo M2 beads for 2 h at 4°C. Following this, SDS–PAGE and immunoblotting were performed as described above. Densitometric quantification was performed using the Image J software and Image Lab Software.

All co-immunoprecipitation experiments were performed at least twice. Statistical significance of immunoblotting data was assessed using a one-way analysis of variance (ANOVA) and a Tukey's honestly significantly different (HSD) post hoc test for multiple comparisons, or unpaired Students t-test for pairwise comparisons. In all cases, P values of <0.05 were considered statistically significant and are indicated with *P < 0.05, **P < 0.01, ***P < 0.005, ****P < 0.001. Statistical analysis was performed in GraphPad Prism.

LLC0150 treatment of PDX-derived CRPC organoids and VCaP xenografts

Patient-derived xenograft (PDX) tumors were resected from animals, minced into small pieces, and incubated with collagenase II (5mg/mL) for 1hr at 37C followed by TrypLE digestion for 10 min. The cell suspension was then filtered, resuspended in Matrigel, and plated as droplets in 48-well plates to allow cells to grow as organoids. The culture conditions were the following: AdDMEM/F12 medium supplemented with HEPES, Glutamax, penicillin/streptomycin, B27, N-Acetylcysteine (1.25mM), recombinant R-spondin-1 (500ng/m), recombinant Noggin (100ng/mL), A83-01 (500nM), recombinant FGF-10(10ng/mL), FGF-2 (5ng/mL), Prostaglandin E2 (1uM), SB2020190, nicotinamide (10mM), DHT (1nM), and Y27632 (10uM). Following 5 days of drug incubation, cell viability was assessed using 3D CellTiter-Glo (Promega G9683).

For the xenograft efficacy experiment, 3 million VCaP cells resuspended in 50% Matrigel (BD Biosciences) + serum-free media solution were subcutaneously injection into the dorsal flanks of CB-17 NOD/SCID mice. Once VCaP tumors grew to ~200 mm^3 in size, mice were randomized into the control and drug arms. 100ul of either the solvent (5% DMSO+ 95%PEG400, n=2) or LLC0150 (resuspended at 40mg/ml, n=4) was injected directly into each tumor for five consecutive days. Four hours after the last dosage, tumors were harvested and processed for western blotting or IHC/IF assays, as described above. All animal studies were approved by the University of Michigan Institutional Animal Care and Use Committee (IACUC).

LLC0150 formula for in vivo studies: LLC0150 was first dissolved in DMSO via sonication. Next, the solution was further mixed with PEG400 and sonicated until completely dissolved. The ratio of DMSO and PEG400 was 5:95, and the final concentration of LLC0150 was 40 mg/mL.

Murine prostate tumor xenografts

Two million 22RV1 expressing Cas9, NSD2 KO, NSD2 rescue, and NSD2dTAG cells were subcutaneously injected into the right flank or both dorsal flanks of 5–8-week-old male non-castrated and castrated NCI/NOD SCID/NCr mice (Charles River, strain code: 561, Jackson Laboratory, strain code: 005557) in 50% Matrigel (Corning, 354234). 22RV1 cells were chosen for *in vivo* experiments due to their higher take rate (almost 100%) as well as faster growth kinetics as xenografts. Thus, the 22RV1 model allowed us to cleanly assess the effect of NSD2 activity on the subcutaneous grafting ability of prostate cancer cells without other confounding technical variables. The tumor measurement study ended at 60 days, and mice with tumors were sacrificed due to body condition or used for subsequent analysis. Tumor tissue was harvested and snap-

frozen in liquid nitrogen for immunoblotting analysis. Mice that did not form tumors at this time continued to be monitored for tumor growth and body weight for first detection of tumor analysis up to 80 days. The chi-square- or t-test was used to evaluate the association of individual tumor characteristics with engraftment.

dTAGV-1 was formulated by dissolving into DMSO and further diluting with 10% solutol (Sigma): 0.9% sterile saline (Moltox) (w:v) with the final formulation containing 5% DMSO. Mice were randomized into groups and IP injections of dTAGV-1 or vehicle were given at 25 mg/kg of body weight every 24 hours. No data points were excluded from any of the animals.

Drug preparations: *in vitro* and *in vivo*

Enzalutamide (Selleckchem, S1250) and dTAG-13 (Sigma, SML2601) were dissolved and aliquoted in DMSO (Sigma, D2650). ARD61 was a kind gift from Dr. Shaomeng Wang. R1881 and UNC6934 were purchased from Sigma Aldrich, and JQ1 and EPZ-6438 were obtained from Selleck Chemicals. LLC0150 (NSD1/2) PROTAC was synthesized in Dr. Ke Ding's lab at the Shanghai Institute of Organic Chemistry, Chinese Academy of Sciences.

Size exclusion chromatography

VCaP/LNCaP nuclear extracts were obtained using NE-PER nuclear extraction kit (Thermo Scientific) and dialyzed against FPLC buffer (20 mM Tris-HCl, 0.2 mM EDTA, 5mM MgCl2, 0.1 M KCl, 10% (v/v) glycerol, 0.5 mM DTT, 1 mM benzamidine, 0.2 m MPMSF, pH7.9). 5mg of nuclear protein was concentrated in 500ul using a Microcon centrifugal filter (Millipore) and then applied to a Superose 6 size exclusion column (10/300 GL GE Healthcare) pre-calibrated using the Gel Filtration HMW Calibration Kit (GE Healthcare). 500 μl elute was collected for each fraction at a flow rate of 0.5ml/min, and eluted fractions were subjected to SDS-PAGE and western blotting.

ChIP-seq data quantification and spike-in controls

The castration-resistant prostate cancer (CRPC), normal, and primary prostate cancer (PCa) dataset fastqs were pulled from GEO from Baca et al and Pomerantz et al5,83 (GSE130408 and GSE70079) and processed using our previously described ChIP-seq pipelines, adapted for single end reads. Picard utility MergeSamFiles was utilized to combine the aligned bam files. Samtools view -bs was used to subsample the combined bam file to a depth of approximately 100M reads for AR datasets and 250M for H3K27ac datasets. Peak calling was then repeated using MACS2 callpeak, and output bedgraphs were converted using wigToBigWig. Motif enrichment ratios between primary PCa (or CRPC) to normal prostate tissues were calculated and plotted in Fig. 2g and Fig. S4e. Normalized read density at specific motifs is plotted in Fig. 2h and Fig. S4g.

Normalized read coverage barplots: ChIP-seq read coverage was calculated using samtools78 on the aligned read files for regions of interest (samtools bedcov) and normalized by total number of reads (samtools view -c -F 260). Statistics were performed using rstatix84 (0.7.0) and tidyverse85 (1.3.1) to calculate a t-test significance. R package ggplot2 (3.3.6) was used to generate the bar plots.

ChIP-seq spike-in: For the histone ChIP-seq samples, spike-in normalization was performed as mentioned in Egan et al. 201686. 2% of the drosophila chromosome was added to the histone ChIP samples before the IP was performed. The reads from these samples were first trimmed using Atria (default options, v4.0.0)87 and then aligned to hg38 (GRCh38) using bowtie2 ("bowtie align" using options –local –no-mixed –discordant ,v2.5.1)88. Reads were then also aligned to the drosophila genome dm6 (BDGP Release 6 + ISO1 MT) and the number of reads mapping to this genome was counted and percentage of drosophila reads in each sample was calculated. Scaling factors for each sample were then calculated by scaling up human reads such that the percentage of drosophila reads in each sample was the same. Using DeepTools89, bigWig files for the samples were then generated using the scaleFactors option while providing respective scaling factors calculated in the previous step.

Supplementary References:

1. Wang, G. G., Cai, L., Pasillas, M. P. & Kamps, M. P. NUP98-NSD1 links H3K36 methylation to Hox-A gene activation and leukaemogenesis. *Nat. Cell Biol.* 9, 804–812 (2007).
2. Malgeri, U. *et al.* Detection of t(4;14)(p16.3;q32) chromosomal translocation in multiple myeloma by reverse transcription-polymerase chain reaction analysis of IGH-MMSET fusion transcripts. *Cancer Res.* 60, 4058–4061 (2000).
3. Chesi, M. *et al.* The t(4;14) translocation in myeloma dysregulates both FGFR3 and a novel gene, MMSET, resulting in IgH/MMSET hybrid transcripts. *Blood* 92, 3025–3034 (1998).
4. Vo, J. N. *et al.* The genetic heterogeneity and drug resistance mechanisms of relapsed refractory multiple myeloma. *Nat. Commun.* 13, 3750 (2022).
5. Jaffe, J. D. *et al.* Global chromatin profiling reveals NSD2 mutations in pediatric acute lymphoblastic leukemia. *Nat. Genet.* 45, 1386–1391 (2013).
6. Li, J. *et al.* NSD2-E1099K Mutation Leads to Glucocorticoid-Resistant B Cell Lymphocytic Leukemia in Mice. *Blood* 136, 3–4 (2020).
7. Kuo, A. J. *et al.* NSD2 links dimethylation of histone H3 at lysine 36 to oncogenic programming. *Mol. Cell* 44, 609–620 (2011).
8. Schmitges, F. W. *et al.* Histone methylation by PRC2 is inhibited by active chromatin marks. *Mol. Cell* 42, 330–341 (2011).
9. Oyer, J. A. *et al.* Point mutation E1099K in MMSET/NSD2 enhances its methyltranferase activity and leads to altered global chromatin methylation in lymphoid malignancies. *Leukemia* 28, 198–201 (2014).
10. Pierro, J. *et al.* The NSD2 p.E1099K Mutation Is Enriched at Relapse and Confers Drug Resistance in a Cell Context-Dependent Manner in Pediatric Acute Lymphoblastic Leukemia. *Mol. Cancer Res.* 18, 1153–1165 (2020).
11. Finogenova, K. *et al.* Structural basis for PRC2 decoding of active histone methylation marks H3K36me2/3. *Elife* 9, (2020).
12. McLean, C. Y. *et al.* GREAT improves functional interpretation of cis-regulatory regions. *Nat. Biotechnol.* 28, 495–501 (2010).
13. Tanigawa, Y., Dyer, E. S. & Bejerano, G. WhichTF is functionally important in your open chromatin data? *PLoS Comput. Biol.* 18, e1010378 (2022).
14. Chen, E. Y. *et al.* Enrichr: interactive and collaborative HTML5 gene list enrichment analysis tool. *BMC Bioinformatics* 14, 128 (2013).

15. Killian, T. & Gatto, L. Exploiting the DepMap cancer dependency data using the depmap R package. *F1000Res.* 10, 416 (2021).
