# Supplementary material for: NSD2 is a requisite subunit of the AR/FOXA1 neo-enhanceosome in promoting prostate tumorigenesis
Source: Nat Genet. 2024 Sep 9;56(10):2132–43. doi: 10.1038/s41588-024-01893-6 (PMC11525188; doi:10.1038/s41588-024-01893-6)
Supplement: Supplementary file 2 — Reporting Summary [file 41588_2024_1893_MOESM2_ESM.pdf]

nature portfolio

Corresponding author(s): Abhijit Parolia, Arul Chinnaiyan, and Irfan Asangani

Last updated by author(s): 07/12/2024

Reporting Summary

Nature Portfolio wishes to improve the reproducibility of the work that we publish. This form provides structure for consistency and transparency in reporting. For further information on Nature Portfolio policies, see our Editorial Policies and the Editorial Policy Checklist.

Statistics

For all statistical analyses, confirm that the following items are present in the figure legend, table legend, main text, or Methods section.

n/a	Confirmed	
☐	☒	The exact sample size (*n*) for each experimental group/condition, given as a discrete number and unit of measurement
☐	☒	A statement on whether measurements were taken from distinct samples or whether the same sample was measured repeatedly
☐	☒	The statistical test(s) used AND whether they are one- or two-sided<br>*Only common tests should be described solely by name; describe more complex techniques in the Methods section.*
☐	☒	A description of all covariates tested
☐	☒	A description of any assumptions or corrections, such as tests of normality and adjustment for multiple comparisons
☐	☒	A full description of the statistical parameters including central tendency (e.g. means) or other basic estimates (e.g. regression coefficient) AND variation (e.g. standard deviation) or associated estimates of uncertainty (e.g. confidence intervals)
☐	☒	For null hypothesis testing, the test statistic (e.g. *F*, *t*, *r*) with confidence intervals, effect sizes, degrees of freedom and *P* value noted<br>*Give P values as exact values whenever suitable.*
☒	☐	For Bayesian analysis, information on the choice of priors and Markov chain Monte Carlo settings
☒	☐	For hierarchical and complex designs, identification of the appropriate level for tests and full reporting of outcomes
☒	☐	Estimates of effect sizes (e.g. Cohen's *d*, Pearson's *r*), indicating how they were calculated

Our web collection on statistics for biologists contains articles on many of the points above.

Software and code

Policy information about availability of computer code

Data collection

No software was used for data collection.

Data analysis

All custom codes used for data analyses are freely available from the following public repositories:
https://github.com/mcieslik-mctp/papy
https://github.com/mcieslik-mctp/hpseq
https://github.com/mcieslik-mctp/bootstrap-rnascape
https://github.com/mcieslik-mctp/codac
https://github.com/mcieslik-mctp/crisp
https://github.com/mcieslik-mctp/
https://github.com/mctp/

Computational tools used:
GraphPad Prism 9 and in-built statistical tools
bcl2fastq conversion software (v2.20)
Pairtools (version 0.3.0)
EdgeR (bulk: edgeR_3.39.6, single cell: v3.36.0)
Limma-Voom (limma_3.53.10)
fgsea (fgsea_1.24.0)
EnhancedVolcano (EnhancedVolcano_1.15.0)
R (R version 4.2.1 70–72)
ChipPeakAnno (version 3.0.0)

ChipSeeker (version 1.29.1)
Sushi (Sushi_1.32.0)
Trimmomatic (version 0.39)
bwa (version 0.7.17-r1198-dirty)
samtools (v1.1)
picard MarkDuplicates (v2.26.0-1-gbaf4d27-SNAPSHOT)
MACS2 (v2.2.7.1)
bedtools (v2.27.1)
UCSC's tool wigtoBigwig (v2.8)
bowtie2 v2.5.1
HOMER (version v.4.10)
Deeptools (v3.5.1)
Kallisto (version 0.46.1)

Webtools used:
GREAT (v4.0.4; from http://great.stanford.edu/public/html/)
Enrichr (from http://amp.pharm.mssm.edu/Enrichr/
XSTREME from the MEME Suite (v 5.5.5; from https://meme-suite.org/meme/doc/xstreme.html?man_type=web)

For manuscripts utilizing custom algorithms or software that are central to the research but not yet described in published literature, software must be made available to editors and reviewers. We strongly encourage code deposition in a community repository (e.g. GitHub). See the Nature Portfolio guidelines for submitting code & software for further information.

Data

Policy information about availability of data

All manuscripts must include a data availability statement. This statement should provide the following information, where applicable:

- Accession codes, unique identifiers, or web links for publicly available datasets
- A description of any restrictions on data availability
- For clinical datasets or third party data, please ensure that the statement adheres to our policy

All data are available in the manuscript or the supplementary information. All materials are available from the authors upon request. All raw next-generation sequencing, including ChIP-seq and RNA-seq, data generated in this study are deposited in the Gene Expression Omnibus (GEO) repository (accession number: GSE242737) at NCBI and is publicly available. Additionally, AR ChIPseq from castration-resistant prostate cancer (CRPC), normal, and primary prostate cancer (PCa) were pulled from GEO repositories from Baca et al and Pomerantz et al (GSE130408 and GSE70079). Public scRNA-seq datasets from primary prostate cancer were downloaded from GEO or a website provided by the authors (GSE193337, GSE185344, www.prostatecellatlas.org). Functional gene sets were pulled from hallmark and C2 MsigDB pathways (/www.gsea-msigdb.org).

Research involving human participants, their data, or biological material

Policy information about studies with human participants or human data. See also policy information about sex, gender (identity/presentation), and sexual orientation and race, ethnicity and racism.

Reporting on sex and gender	All patient samples used in this study are from the University of Michigan pathological archives. All the specimens are from male patients given that the study is focused on prostate cancer.
Reporting on race, ethnicity, or other socially relevant groupings	All samples were from White, Non-Hispanic patients at the University of Michigan.
Population characteristics	N/A
Recruitment	Patient tissues from biopsies of prostate tumors were acquired from the University of Michigan pathology archives.
Ethics oversight	Use of clinical formalin-fixed paraffin embedded specimens from the archives was approved by the University of Michigan Institutional Review Board and does not require patient consent.

Note that full information on the approval of the study protocol must also be provided in the manuscript.

Field-specific reporting

Please select the one below that is the best fit for your research. If you are not sure, read the appropriate sections before making your selection.

☒ Life sciences ☐ Behavioural & social sciences ☐ Ecological, evolutionary & environmental sciences

For a reference copy of the document with all sections, see nature.com/documents/nr-reporting-summary-flat.pdf

Life sciences study design

All studies must disclose on these points even when the disclosure is negative.

Sample size	All sample size details for the analyses carried out in this study are reported in the Methods section and/or figure legends. No statistical methods were used to predetermine sample sizes. Sample sizes were based on prior research experience of similar assays rather than a power analyses.
Data exclusions	No data was excluded from the published publicly-available patient sequencing studies. For biologically experiments, no data exclusions were made. For the in vivo studies, we have used 5-7 mice bearing 10-14 tumors in each group to allow for statistical assessments.
Replication	All in vitro experiments were independently repeated at least three times, with all replication attempts producing similar results. Reproducibility between RNAseq and ChIPseq samples was assessed on normalized alignment files using principal component analysis, unsupervised hierarchal clustering, or correlation analyses with good reproducibility observed across replications.
Randomization	For animal studies, mice were randomly assigned to treatment groups. For all other in vitro experiments, we used a common cell suspension to plate for both control and treatment groups.
Blinding	All histo-pathological evaluations of tissues and IHC/staining-based scoring for drug toxicity studies were carried out in a blinded manner by two independent pathologists. For all other experiments, the analyses did not require blinding as data quantification was carried out using instruments and automated workflows with no manual steps.

Reporting for specific materials, systems and methods

We require information from authors about some types of materials, experimental systems and methods used in many studies. Here, indicate whether each material, system or method listed is relevant to your study. If you are not sure if a list item applies to your research, read the appropriate section before selecting a response.

Materials & experimental systems

n/a	Involved in the study	
☐	☒	Antibodies
☐	☒	Eukaryotic cell lines
☒	☐	Palaeontology and archaeology
☐	☒	Animals and other organisms
☒	☐	Clinical data
☒	☐	Dual use research of concern
☒	☐	Plants

Methods

n/a	Involved in the study	
☐	☒	ChIP-seq
☒	☐	Flow cytometry
☒	☐	MRI-based neuroimaging

Antibodies

Antibodies used

Target antigen; Vendor; Catalog number; Lot number; Application
NSD1 (NeuroMab: 75-280, Clone#N312/10; Western Blot 1:500);
NSD2 (Abcam:ab75359, Clone#29D1, Western Blot 1:1000, Immunofluorescence 1:200);
NSD3(Cell Signaling Technologies: 92056S, Clone#D4N9N, Western Blot 1:1000);
KLK3/PSA (Dako:A0562, Lot: 00093790, Western Blot 1:2000),
FKBP5(Cell Signaling Technologies: 12210,Clone#: D5G2, Western Blot 1:1000),
NKX3-1(Cell Signaling Technologies:83700S, clone#: D2Y1A, Western Blot 1:1000),
FOXA1 C-terminal (Thermo Fisher Scientific: PA5-27157, Lot# VFS004672A, Western Blot 1:1000 and ChIP-seq 2ug/4M cells);
AR (Millipore: 06-680, Western blot 1:1000 and ChIP-seq 2ug/4M cells));
AR (Abcam: ab133273, Clone#: EPR1535(2), Western blot 1:1000);
H3 (Cell Signaling Technologies: 3638S, Clone#: 96C10, Western blot 1:2000);
GAPDH (Cell Signaling Technologies: 3683, Clone#: 14C10, Western blot) 1:2000;
H3K27me3(Millipore: 07-449, Western blot 1:2000 and ChIP-seq 1ug/2M cells);
H3K36me2 (Cell Signaling Technologies: 2901S, Western blot 1:2000);
H3K27Ac (Active Motif, Cat#39336; ChIP-seq 1ug/2M cells);
H3K4me1 (Abcam: ab8895; ChIP-seq 1ug/2M cells);
H3K4me2 (CST: C64G9; Clone#: C64G9, ChIP-seq 1ug/2M cells);
H3K36me2 (Abcam: ab9049; ChIP-seq 1ug/2M cells);
Phospho-AR (Ser-81) (Millipore, Cat# 07-1375-EMD, Western Blot 1:1000),
HALO (Promega ,Cat# G9281, Western Blot 1:1000 and co-immunopreciptation),
HA (Cell Signaling Technologies, Cat# 3724S, Clone#: C29F4, Western Blot 1:1000 and co-immunopreciptation),
His (Cell Signaling Technologies, Cat#2365,Western Blot 1:1000 and co-immunopreciptation)
CK8 (Abcam, epitope: Clone#: EP1628Y, Cat# ab53280, Immunofluorescence 1:200)

Validation

All antibodies used in this study are from reputed commercial vendors and have been validated by the vendors (see website). QC data is directly available from all the vendor listed above and these antibodies have been commonly used in other publications.
NSD1, https://www.antibodiesinc.com/products/anti-nsd1-antibody-n312-10-75-280

Manufacturer states that the antibody "is produced in-house from hybridoma clone N312/10. It detects human and mouse NSD1, and is purified by Protein A chromatography." Also, "[antibody] does not cross-react with NSD2 or NSD3. Each new lot of antibody is quality control tested on cells overexpressing target protein and confirmed to give the expected staining pattern."

NSD2, https://www.abcam.com/products/primary-antibodies/whsc1nsd2-antibody-29d1-ab75359.html. Manufacturer states, its "suitable for IHC, IP, WB and reacts with human samples."

NSD3, https://www.cellsignal.com/products/primary-antibodies/whsc1l1-d4n9n-rabbit-mab/92056. Manufacturer: "Monoclonal antibody is produced by immunizing animals with a synthetic peptide corresponding to residues surrounding Pro117 of human WHSC1L1 protein. It recognizes endogenous levels of total WHSC1L1 protein, both long and short isoforms."

KLK3/PSA, https://www.citeab.com/antibodies/3382929-a0562-prostate-specific-antigen-psa. We have validated this antibody in our lab by treating LNCaP and VCaP cells with AR antagonistic drugs that led to a marked decrease in KLK3/PSA levels. We have used it in numerous publications from our group.

FKBP5, https://www.cellsignal.com/products/primary-antibodies/fkbp5-d5g2-rabbit-mab/12210. Manufacturer: "Monoclonal antibody is produced by immunizing animals with a synthetic peptide corresponding to residues surrounding Arg222 of human FKBP5 protein. This antibody does not cross-react with FKBP4 protein."

NKX3-1, https://www.cellsignal.com/products/primary-antibodies/nkx3-1-d2y1a-xp-rabbit-mab/83700. Manufacturer states this antibody is reactive to human NKX3.1 and its specificity was confirmed by running a blot with prostate cancer cells (positive control) and DND-41 cells that are negative for NKX3.1 expression.

FOXA1, https://www.thermofisher.com/antibody/product/FOXA1-Antibody-Polyclonal/PA5-27157. Manufacturer: "This Antibody was verified by Knockdown to ensure that the antibody binds to the antigen stated."

AR Millipore, https://www.emdmillipore.com/US/en/product/Anti-Androgen-Receptor-Antibody,MM_NF-06-680. Manufacturer: "This antibody recognizes the Modulation Region within the N-terminus of Androgen Receptor." It was validated by western blotting in LNCaP cells.

AR abcam, https://www.abcam.com/products/primary-antibodies/androgen-receptor-antibody-epr15352-ab133273.html. Manufacturer states that this antibody reacts with Mouse, Rat, Human samples.

H3, https://www.cellsignal.com/products/primary-antibodies/histone-h3-96c10-mouse-mab/3638. Manufacturer states that this antibody "detects endogenous levels of total Histone H3 protein, including isoforms H3.1, H3.2, and H3.3. The antibody does not cross-react with other histone proteins, including the Histone H3 variant CENP-A." It has been validated using western blotting.

GAPDH, https://www.cellsignal.com/products/antibody-conjugates/gapdh-14c10-rabbit-mab-hrp-conjugate/3683. Manufacturer states it reacts with human, mouse, rat and monkey GAPDH and has been validated using western blotting.

H3K27me3, https://www.emdmillipore.com/US/en/product/Anti-trimethyl-Histone-H3-Lys27-Antibody,MM_NF-07-449?ReferrerURL=https%3A%2F%2Fwww.google.com%2F. Manufacturer: "[Antibody is] routinely evaluated by western blot in acid extracted proteins from HeLa cells."

H3K36me2, https://www.cellsignal.com/products/primary-antibodies/di-methyl-histone-h3-lys36-c75h12-rabbit-mab/2901. Manufacturer states that this antibody "detects endogenous levels of histone H3.1, histone H3.2, and histone H3.3, only when di-methylated on Lys36. The antibody does not cross-react with non-methylated, mono-methylated, or tri-methylated Lys36. In addition, the antibody does not cross-react with di-methylated histone H3 Lys4, Lys9, Lys27, Lys79 or di-methylated histone H4 Lys20." It has been validated by western blotting.

H3K27Ac , https://www.activemotif.com/catalog/details/39135/histone-h3-acetyl-lys27-antibody-pab-1. Manufacturer states that the antibody has been tested by Western blot as well as the dot blot analysis"

Phospho-AR (Ser-81) ,https://www.emdmillipore.com/US/en/product/Anti-phospho-Androgen-Receptor-Ser81-Antibody,MM_NF-07-1375. Manufacturer states, " Detects Androgen Receptor (AR) only when phosphorylated on Ser81" and is reactive only to human AR. It has been validated by running western blots on LNCaP lysates.

HALO, https://www.promega.com/products/protein-detection/primary-and-secondary-antibodies/anti-halotag-pab/?catNum=G9281. Manufacturer: " The antibody is purified using Protein G affinity resin and supplied at 1mg/ml in PBS. The antibody detects HaloTag® fusion proteins in Western blot hybridization and immunocytochemistry applications with high sensitivity and specificity."

HA, https://www.cellsignal.com/products/primary-antibodies/ha-tag-c29f4-rabbit-mab/3724. Manufacturer: The antibody was validated using "western blot analysis of extracts from HeLa cells untransfected or transfected with either HA-FoxO4 or HA-Akt3 plasmids. The antibody may cross-react with a protein of unknown origin ~100kDa."

His, https://www.cellsignal.com/products/images/2365_ific_jp.jpg6. Manufacturer: the antibody was validated using "western blot analysis of extracts from cells expressing C-terminal His-tagged protein or control extract. It detects recombinant proteins containing the 6xHis epitope tag. The antibody recognizes the 6xHis-tag fused to either the amino or carboxy terminus of targeted proteins in transfected cells. The antibody may cross-react with a protein of unknown origin ~60-70kDa."

H3K4me1 (Abcam: ab8895), https://www.abcam.com/products/primary-antibodies/histone-h3-mono-methyl-k4-antibody-chip-grade-ab8895.html Manufacturer: The anitbody is suitable for IHC-P, ICC/IF, ChIP, WB and reacts with Human, Mouse, Rat, Cow samples.

H3K4me2 (CST: C64G9), https://www.cellsignal.com/products/primary-antibodies/di-methyl-histone-h3-lys4-c64g9-rabbit-

mab/9725. Manufacturer: The antibody has been validated using "western blot analysis of whole cell lysates from HeLa, NIH/3T3, C6 and COS cells. It detects endogenous levels of histone H3 when di-methylated on Lys4. This antibody shows weak cross-reactivity with histone H3 that is mono-methylated on Lys4 but does not cross-react with non-methylated or tri-methylated histone H3 Lys4. In addition, the antibody does not cross-react with methylated histone H3 Lys9, Lys27, Lys36 or histone H4 Lys20."

CK8 (Abcam: ab53280): https://www.abcam.com/en-us/products/primary-antibodies/cytokeratin-8-antibody-ep1628y-cytoskeleton-marker-ab53280. Manufacturer: "Antibody is suitable for IP, WB, IHC-Fr, ICC/IF, IHC-P and reacts with Human, Mouse, Rat samples."

Eukaryotic cell lines

Policy information about cell lines and Sex and Gender in Research

Cell line source(s)

Most cell lines were originally obtained from ATCC, DSMZ, ECACC, or internal stock. All the cells were genotyped to confirm their identity at the University of Michigan Sequencing Core and tested routinely for Mycoplasma contamination. Additionally, all the cell lines were genotyped every two months to confirm their identity. Cells were grown media conditions prescribed by ATCC, DSMZ or ECACC.

Here is the list of all the cell lines used in this study:
RS4;11
MOLT3
RPMI-8402
MM1.S
H1048
MM1.R
TC-205
H1836
TC32
MOLT4
DoHH2
CHLA-9
CHLA-258
SUM185PE
NB-1643
CB-AGPN
LASCPC-01
CAMA-1
SEM
R-CHACV
T47D
VCaP
MDA-PCa-2b
KARPAS-25
COG-N-561
CHLA-218
MDA-MB-330
H446
PA-1
MFM-223
WA-72-PS
22RV1
LNCaP
MDA-MB-468
CWRR1
MDA-MB-453
N87
IMR90
WA-72-As
COG-E-352
H524
NTERA-2
LAPC4
ZR-75-1
CHLA-99
CHLA-25
Caov-3
MEG-01
BEAS-2B
COLO205
MDA-MB-231
MDA-MB-436
5637
H211
SAOS-2

HCC1143
HCC1146
TC-106
TC-138
SW626
HeLa
HCC1187
MDA-MB-415
957/hTERT
COG-N-557
SNU1079
SNU387
BJ
HK2
PC3
RPB1293
H69
RWPE1
A673
TE6
SK-N-MC
HEK293FT
SCaBER
UM-UC3
PrECs
OC-8
HCC1428
RPB1292
SNU16
SNU423
HEPG2
PNT2
KATO III
SK-OV-3
PLC/PRF/5
COG-N-529
MCF7
RPMI8226
K562
DU145
786-O
AGS
SNU840
MCF10A
RWPE2-W99
HT115
CADO-ES-1
SNU-5
DAN-G
SK-HEP-1
LHSAR
SK-MEL-5
WPMY1
ACHN
U2OS
TC-71
H716
LAMA87
A549

Authentication

All cell lines were biweekly tested to be free of mycoplasma contamination and genotyped every month at the University of Michigan Sequencing Core using Profiler Plus (Applied Biosystems) and compared with corresponding short tandem repeat (STR) profiles in the ATCC database to authenticate their identity in culture between passages and experiments.

Mycoplasma contamination

All cells were biweekly tested for mycoplasma contamination using the MycoAlert PLUS Mycoplasma Detection Kit (Lonza) and were found to be continually negative. More details are included in the Methods section

Commonly misidentified lines
(See ICLAC register)

None

Animals and other research organisms

Policy information about studies involving animals; ARRIVE guidelines recommended for reporting animal research, and Sex and Gender in Research

Laboratory animals	NOD/SCID mice were obtained from commercial sources. All mice were housed in a pathogen-free animal barrier facility and all in vivo experiments were initiated with male mice aged 5-8 weeks. All mice were maintained under the conditions of pathogen-free, 12 hours light/12 hours dark cycle, temperatures of 18-23°C, and 40-60% humidity.
Wild animals	No wild animals were used in the study.
Reporting on sex	Male animals were used since prostate cancer is specific to males.
Field-collected samples	No field collected samples were used in the study.
Ethics oversight	The Institutional Animal Care & Use Committee (IACUC) ensures that the highest animal welfare standards are maintained along with the conduct of accurate, valid scientific research through the supervision, coordination, training, guidance, and review of every project proposed to include the use of vertebrate animals at the University of Michigan and the University of Pennsylvania.

Note that full information on the approval of the study protocol must also be provided in the manuscript.

Plants

Seed stocks	Not applicable
Novel plant genotypes	Not applicable
Authentication	Not applicable.

ChIP-seq

Data deposition

☒ Confirm that both raw and final processed data have been deposited in a public database such as GEO.

☒ Confirm that you have deposited or provided access to graph files (e.g. BED files) for the called peaks.

Data access links
May remain private before publication.

All raw next-generation sequencing, including ChIP-seq and RNA-seq, data generated in this study are deposited in the Gene Expression Omnibus (GEO) repository (accession number: GSE242737) at NCBI. The following secure token has been created to allow review of record GSE242737 while it remains in private status: uzmjkswedlmtbmb.

Files in database submission

These NGS fastq files will be deposited as part of this study to GEO:

RNASeq:
LNCaP_sgNCx3_rep1_R1.fq.gz
LNCaP_sgNCx3_rep2_R1.fq.gz
LNCaP_sgNSD1_rep1_R1.fq.gz
LNCaP_sgNSD1_rep2_R1.fq.gz
LNCaP_sgNSD3_rep1_R1.fq.gz
LNCaP_sgNSD3_rep2_R1.fq.gz
VCaP_siNC_DMSO_Rep1_R1.fq.gz
VCaP_siNC_DMSO_Rep2_R1.fq.gz
VCaP_siNC_EPZ6438_72h_Rep1_R1.fq.gz
VCaP_siNC_EPZ6438_72h_Rep2_R1.fq.gz
VCaP_siNSD1_DMSO_Rep1_R1.fq.gz
VCaP_siNSD1_DMSO_Rep2_R1.fq.gz
VCaP_siNSD1and2_DMSO_Rep1_R1.fq.gz
VCaP_siNSD1and2_DMSO_Rep2_R1.fq.gz
VCaP_siNSD2_DMSO_Rep1_R1.fq.gz
VCaP_siNSD2_DMSO_Rep2_R1.fq.gz
LNCaP_DMSO_rep1_R1.fq.gz
LNCaP_DMSO_rep2_R1.fq.gz
LNCaP_LLC0150_24hr_rep1_R1.fq.gz
LNCaP_LLC0150_24hr_rep2_R1.fq.gz
LNCaP_sgNCx3_rep1_R2.fq.gz
LNCaP_sgNCx3_rep2_R2.fq.gz
LNCaP_sgNSD1_rep1_R2.fq.gz
LNCaP_sgNSD1_rep2_R2.fq.gz
LNCaP_sgNSD3_rep1_R2.fq.gz
LNCaP_sgNSD3_rep2_R2.fq.gz

VCaP_siNC_DMSO_Rep1_R2.fq.gz
VCaP_siNC_DMSO_Rep2_R2.fq.gz
VCaP_siNC_EPZ6438_72h_Rep1_R2.fq.gz
VCaP_siNC_EPZ6438_72h_Rep2_R2.fq.gz
VCaP_siNSD1_DMSO_Rep1_R2.fq.gz
VCaP_siNSD1_DMSO_Rep2_R2.fq.gz
VCaP_siNSD1and2_DMSO_Rep1_R2.fq.gz
VCaP_siNSD1and2_DMSO_Rep2_R2.fq.gz
VCaP_siNSD2_DMSO_Rep1_R2.fq.gz
VCaP_siNSD2_DMSO_Rep2_R2.fq.gz
LNCaP_DMSO_rep1_R2.fq.gz
LNCaP_DMSO_rep2_R2.fq.gz
LNCaP_LLC0150_24hr_rep1_R2.fq.gz
LNCaP_LLC0150_24hr_rep2_R2.fq.gz

ChIPSeq:
LNCaP_AR_NSD2KO_R1.fq.gz
LNCaP_AR_NSD2WT_R1.fq.gz
LNCaP_FOXA1_NSD2KO_R1.fastq.gz
LNCaP_FOXA1_NSD2WT_R1.fastq.gz
LNCaP_H3k27Ac_NSD2KO_R1.fq.gz
LNCaP_H3k27Ac_NSD2WT_R1.fq.gz
LNCaP_AR_DMSO_R1.fastq.gz
LNCaP_AR_LLC0150_R1.fastq.gz
LNCaP_FOXA1_DMSO_R1.fastq.gz
LNCaP_FOXA1_LLC0150_R1.fastq.gz
LNCaP_H3K27Ac_DMSO_R1.fastq.gz
LNCaP_H3K27Ac_LLC0150_R1.fastq.gz
LNCaP_AR_NSD2KO_R2.fq.gz
LNCaP_AR_NSD2WT_R2.fq.gz
LNCaP_FOXA1_NSD2KO_R2.fastq.gz
LNCaP_FOXA1_NSD2WT_R2.fastq.gz
LNCaP_H3k27Ac_NSD2KO_R2.fq.gz
LNCaP_H3k27Ac_NSD2WT_R2.fq.gz
LNCaP_AR_DMSO_R2.fastq.gz
LNCaP_AR_LLC0150_R2.fastq.gz
LNCaP_FOXA1_DMSO_R2.fastq.gz
LNCaP_FOXA1_LLC0150_R2.fastq.gz
LNCaP_H3K27Ac_DMSO_R2.fastq.gz
LNCaP_H3K27Ac_LLC0150_R2.fastq.gz

Genome browser session (e.g. UCSC)

N/A

Methodology

Replicates

Multiple biological as well as technical replicates are included.

Sequencing depth

ChIPseq: Sequenced to 50-70M total reads, paired-end mode, 125bp read lengths. Over 97% of uniquely mapped reads.
RNAseq: Sequenced to 25-30M total reads, paired-end mode, 125bp read lengths. Over 97% of uniquely mapped reads.

Antibodies

Antibodies used for ChIP-seqs have been mentioned in the methods section and antibody list above.

Peak calling parameters

MACS2 (Version 2.1.1.20160309) callpeak was used for performing peak calling with the following option: ‘macs2 callpeak–call-summits–verbose 3 -g hs -f BAM -n OUT–qvalue 0.05’. For H3K27ac data, the broad option was used.

Data quality

FastQC was used to quality check the raw sequencing data using standard metrics and default thresholds.

Software

Using deepTools (version 3.3.1) bamCoverage, a coverage file (bigWig format) for each sample was created. The coverage was calculated as the number of reads per bin, where bins are short consecutive counting windows. While creating the coverage file, the data was normalized with respect to each library size. ChIP peak profile plots and read-density heat maps were generated using deepTools, and cistrome overlap analyses were carried out using the ChIPpeakAnno (version 3.0.0) or ChIPseeker (version 1.29.1) packages in R (version 3.6.0).
