# Supplementary material for: NSD2 is a requisite subunit of the AR/FOXA1 neo-enhanceosome in promoting prostate tumorigenesis
Source: Nat Genet. 2024 Sep 9;56(10):2132–43. doi: 10.1038/s41588-024-01893-6 (PMC11525188; doi:10.1038/s41588-024-01893-6)

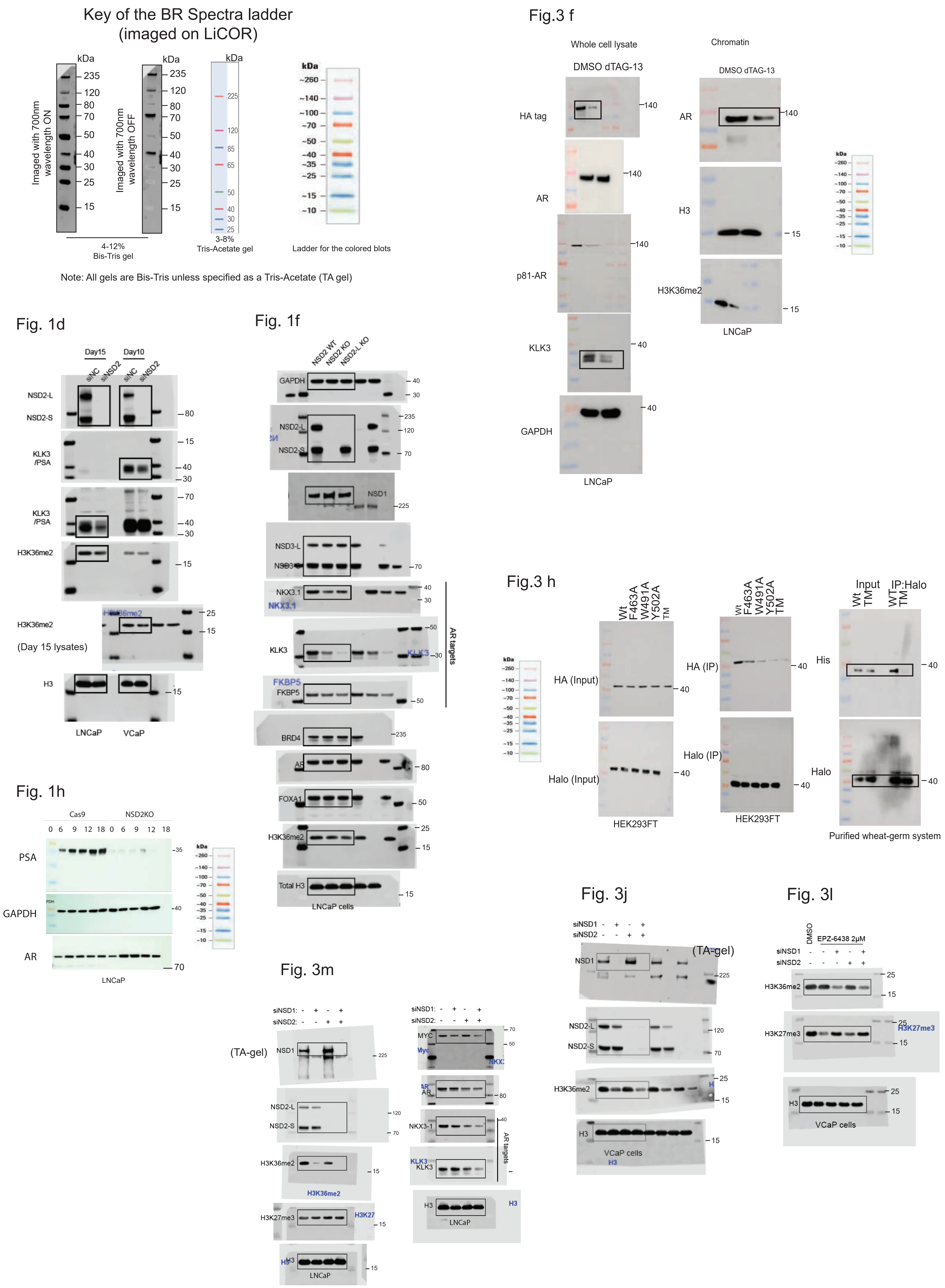

Key of the BR Spectra ladder
(imaged on LiCOR)
Imaged with 700nm wavelength ON
Imaged with 700nm wavelength OFF
4-12%
Bis-Tris gel
3-8%
Tris-Acetate gel
Ladder for the colored blots
Note: All gels are Bis-Tris unless specified as a Tris-Acetate (TA gel)
Fig.3 f
Whole cell lysate
Chromatin
DMSO dTAG-13
HA tag
AR
p81-AR
KLK3
GAPDH
H3
H3K36me2
LNCaP
Fig. 1d
Day15
Day10
siNC
siNSD2
NSD2-L
NSD2-S
KLK3
/PSA
H3K36me2
(Day 15 lysates)
H3
LNCaP
VCaP
Fig. 1f
NSD2 WT
NSD2 KO
NSD2-L KO
GAPDH
NSD1
NSD3-L
NSD3-S
NKX3.1
KLK3
FKBP5
AR targets
BRD4
AR
FOXA1
Total H3
LNCaP cells
Fig.3 h
Wt
F463A
W491A
Y502A
TM
Input
IP:Halo
HA (Input)
HA (IP)
His
Halo (Input)
Halo (IP)
Halo
HEK293FT
Purified wheat-germ system
Fig. 1h
Cas9
NSD2KO
PSA
GAPDH
AR
LNCaP
Fig. 3j
siNSD1
siNSD2
(TA-gel)
VCaP cells
Fig. 3l
DMSO
EPZ-6438 2μM
H3K27me3
VCaP cells
Fig. 3m
MYC
NKX3-1

Fig. 4b

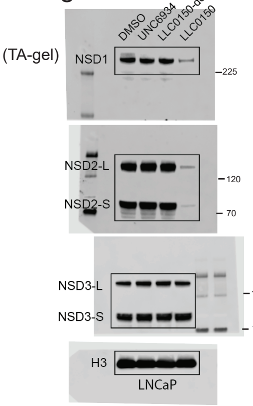

Fig. 4c

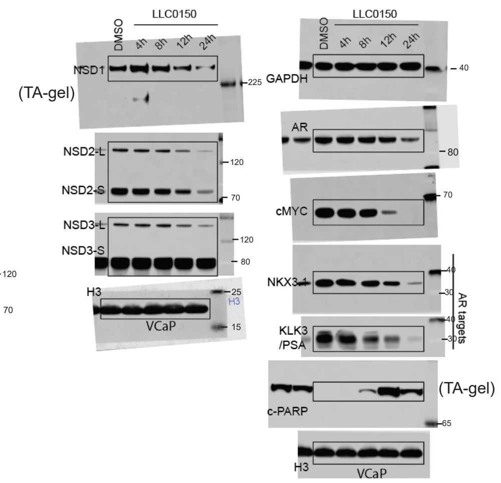

ED Fig. 1e

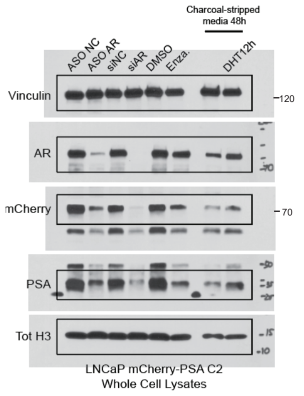

Please note that ED Fig. 1e are radiographic blots and thus the molecular weight ladders are hand noted.
Note: mCherry in the above plots is running at ~70 due to a P2A in-frame fusion with PSA.

ED Fig. 1g

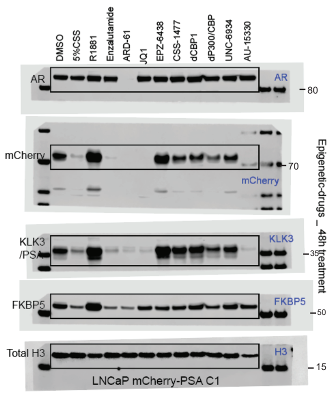

ED Fig. 2a

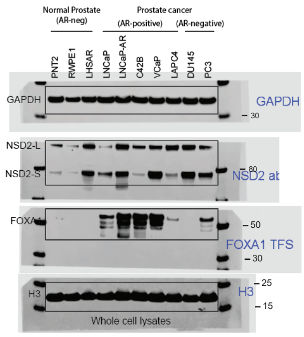

ED Fig. 2d

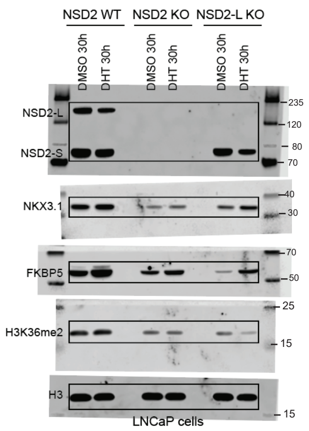

ED Fig. 5a (Top)

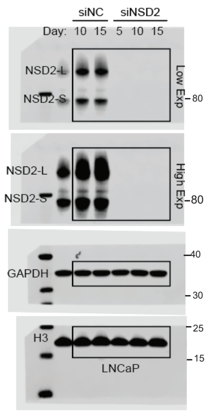

ED Fig. 5b

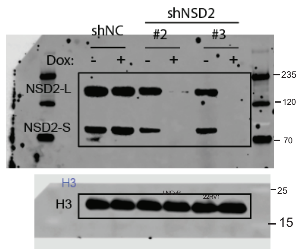

ED Fig. 5d

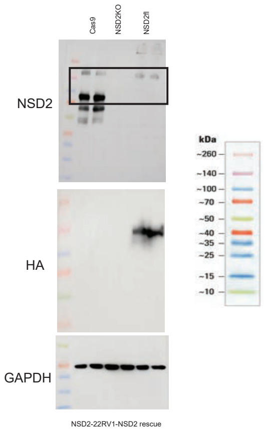

ED Fig. 5a (Bottom)

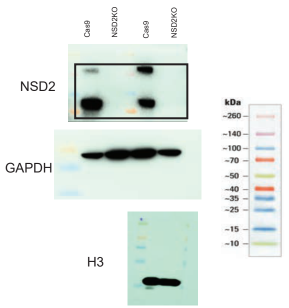

ED Fig. 5e

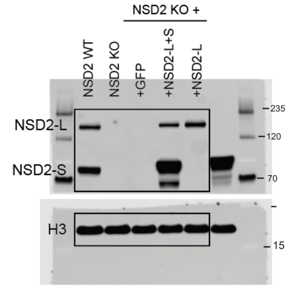

ED Fig. 5f

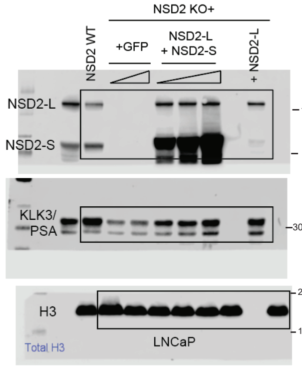

ED Fig. 5g

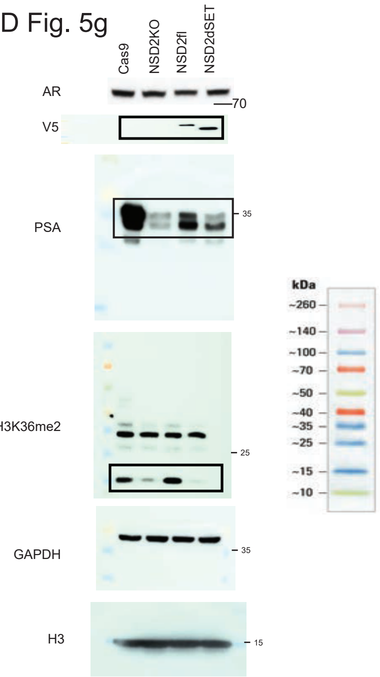

ED Fig. 5h

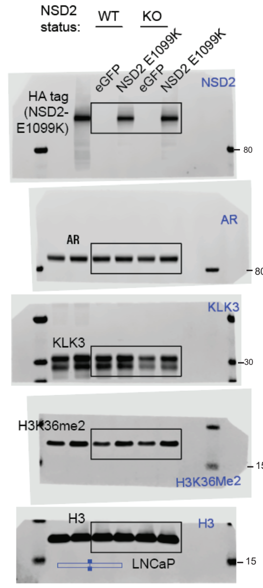

ED Fig. 5i

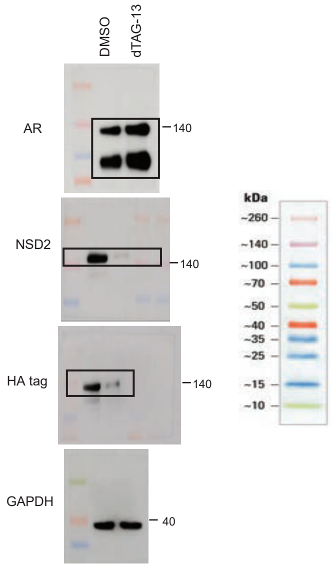

ED Fig. 7a

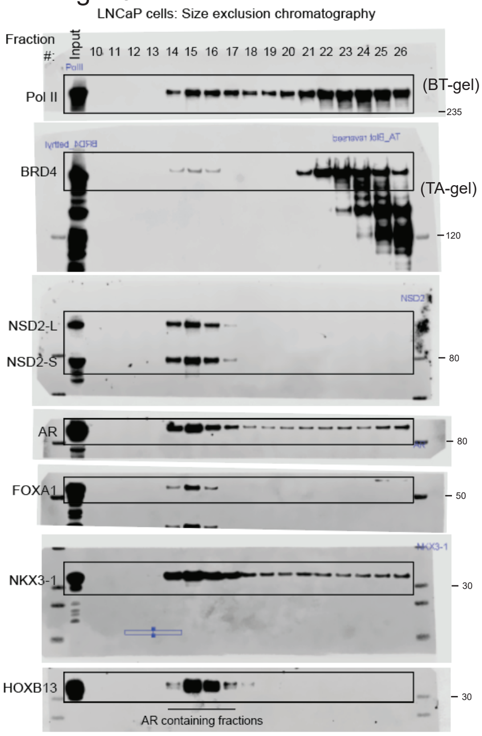

ED Fig. 7b

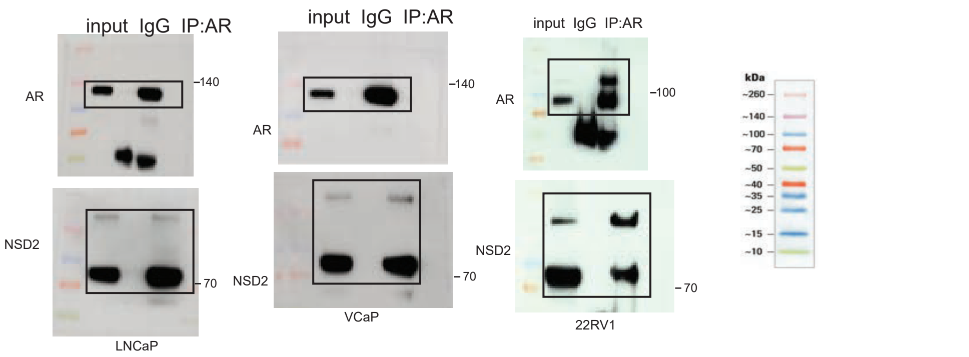

ED Fig. 7c (Left)

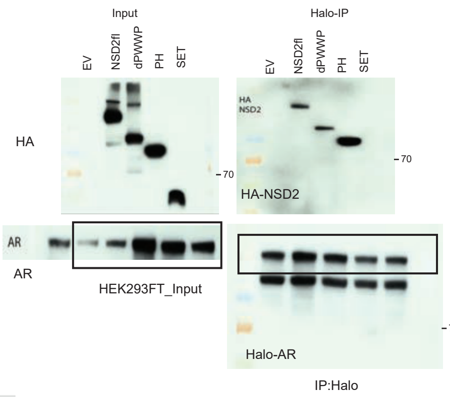

ED Fig. 7c (Right)

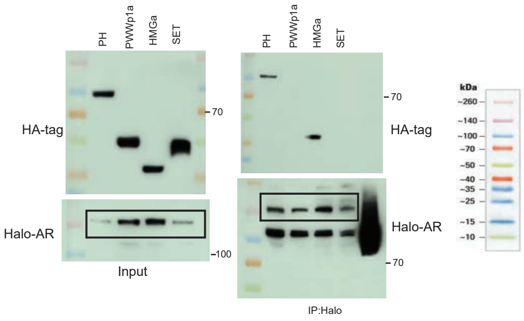

ED Fig. 7g

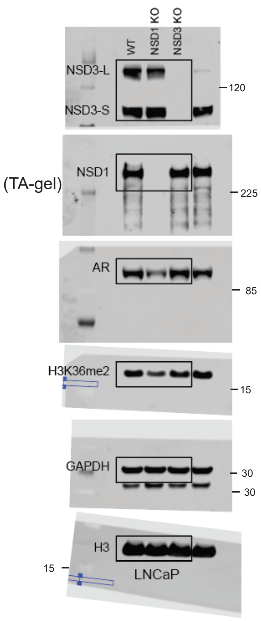

ED Fig. 7i

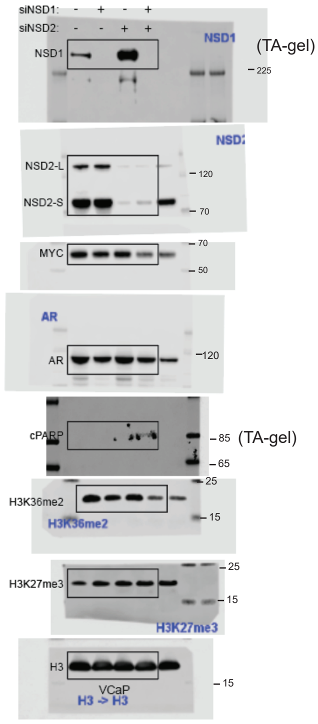

ED Fig. 7d

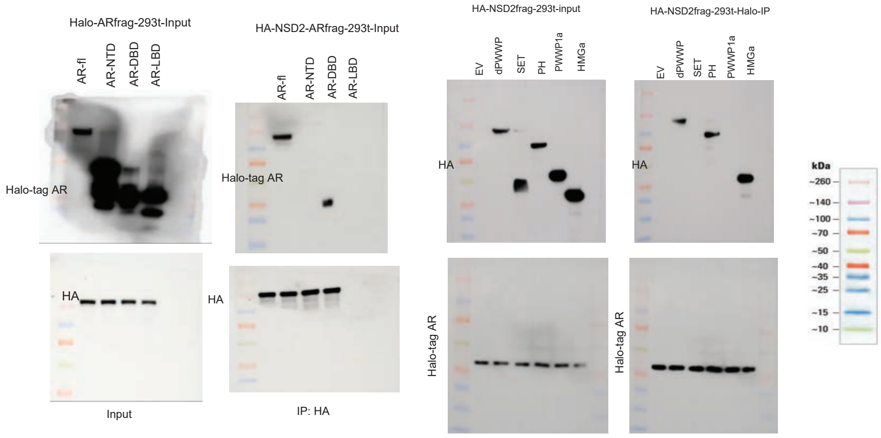

ED Fig. 8a

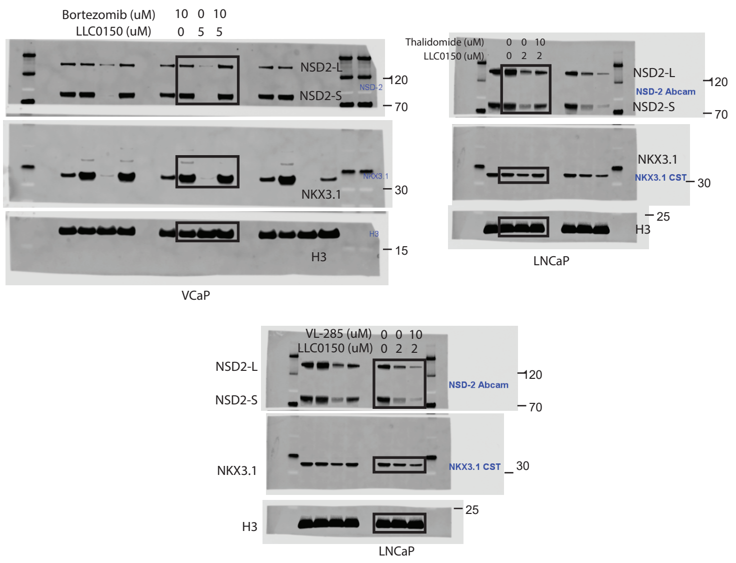

ED Fig. 8d

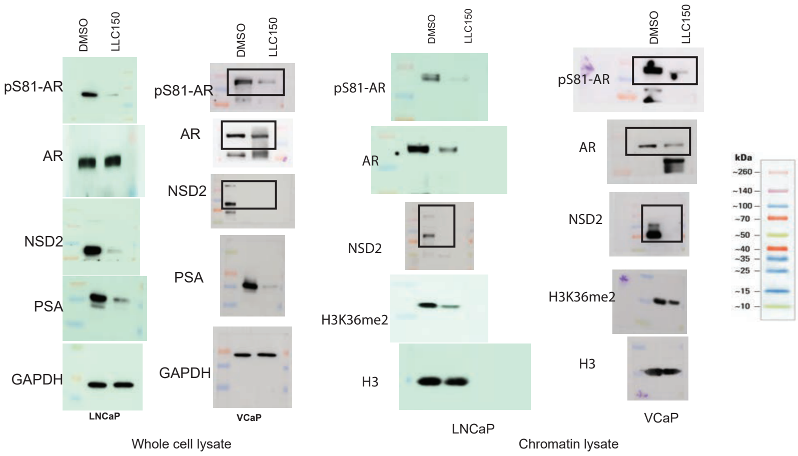

ED Fig. 9b

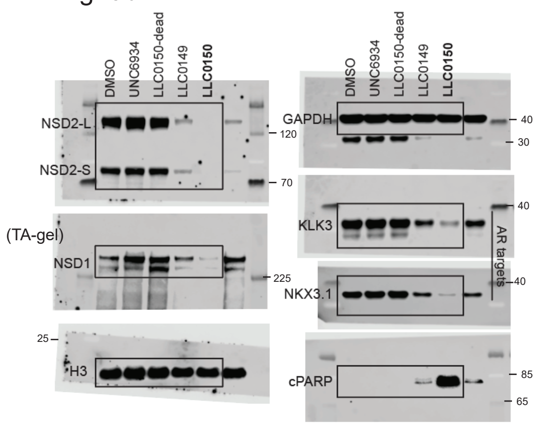

LNCaP cells - 2mM for 72h

ED Fig. 10b

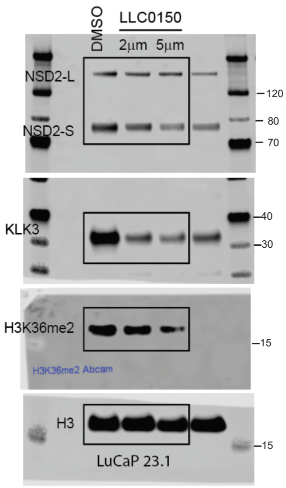

ED Fig. 10g

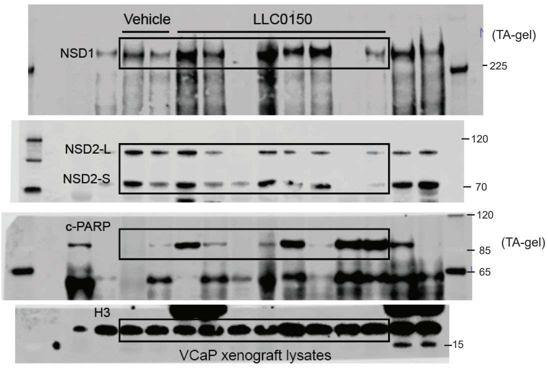

Supplement: Supplementary file 9 — Unprocessed western blots. [file 41588_2024_1893_MOESM9_ESM.pdf]
